# Sublingual Immunization with M2-Based Vaccine Induces Broad Protective Immunity against Influenza

**DOI:** 10.1371/journal.pone.0027953

**Published:** 2011-11-30

**Authors:** Byoung-Shik Shim, Young Ki Choi, Cheol-Heui Yun, Eu-Gene Lee, Yoon Seong Jeon, Sung-Moo Park, In Su Cheon, Dong-Hyun Joo, Chung Hwan Cho, Min-Suk Song, Sang-Uk Seo, Young-Ho Byun, Hae-Jung Park, Haryoung Poo, Baik Lin Seong, Jae Ouk Kim, Huan Huu Nguyen, Konrad Stadler, Dong Wook Kim, Kee-Jong Hong, Cecil Czerkinsky, Man Ki Song

**Affiliations:** 1 Laboratory Science Division, International Vaccine Institute, Seoul, Republic of Korea; 2 Department of Agricultural Biotechnology, Research Institute for Agriculture and Life Sciences, Center for Agricultural Biomaterials, Seoul National University, Seoul, Republic of Korea; 3 College of Medicine and Medical Research Institute, Chungbuk National University, Cheongju, Republic of Korea; 4 Department of Biotechnology, College of Engineering, Yonsei University, Seoul, Republic of Korea; 5 Korean Research Institute of Bioscience and Biotechnology, Daejeon, Republic of Korea; 6 Department of Pharmacy, College of Pharmacy, Hanyang University, Kyeonggi-do, Republic of Korea; 7 Division of Influenza Viruses, Korea National Institute of Health, Osong, Republic of Korea; Hallym University, Republic of Korea

## Abstract

**Background:**

The ectodomain of matrix protein 2 (M2e) of influenza A virus is a rationale target antigen candidate for the development of a universal vaccine against influenza as M2e undergoes little sequence variation amongst human influenza A strains. Vaccine-induced M2e-specific antibodies (Abs) have been shown to display significant cross-protective activity in animal models. M2e-based vaccine constructs have been shown to be more protective when administered by the intranasal (i.n.) route than after parenteral injection. However, i.n. administration of vaccines poses rare but serious safety issues associated with retrograde passage of inhaled antigens and adjuvants through the olfactory epithelium. In this study, we examined whether the sublingual (s.l.) route could serve as a safe and effective alternative mucosal delivery route for administering a prototype M2e-based vaccine. The mechanism whereby s.l. immunization with M2e vaccine candidate induces broad protection against infection with different influenza virus subtypes was explored.

**Methods and Results:**

A recombinant M2 protein with three tandem copies of the M2e (3M2eC) was expressed in *Escherichia coli*. Parenteral immunizations of mice with 3M2eC induced high levels of M2e-specific serum Abs but failed to provide complete protection against lethal challenge with influenza virus. In contrast, s.l. immunization with 3M2eC was superior for inducing protection in mice. In the latter animals, protection was associated with specific Ab responses in the lungs.

**Conclusions:**

The results demonstrate that s.l. immunization with 3M2eC vaccine induced airway mucosal immune responses along with broad cross-protective immunity to influenza. These findings may contribute to the understanding of the M2-based vaccine approach to control epidemic and pandemic influenza infections.

## Introduction

Current seasonal influenza virus vaccines are composed of antigenic determinants from three virus strains, two influenza A virus subtypes (H1N1 and H3N2) and one influenza B virus strain, that are predicted to cause disease during the upcoming influenza season. The feature of the vaccines is to induce neutralizing antibodies (Abs) against the two major viral glycoproteins, the hemagglutinin (HA) and neuraminidase (NA) that undergo frequent antigenic variations [1]. Since the efficacy of these vaccines is by and large strain-specific and hence relatively weak against antigenic variants [2], it is necessary to revaccinate with the updated strains every year. This has led to efforts to develop a universal vaccine capable of inducing protection against different influenza virus subtypes [3].

M2 is a transmembrane protein containing 97 amino acids and the native protein is a homotetramer linked by two disulfide linked dimers [4]. The tetrameric M2 protein forms a proton channel and plays an important role in uncoating the virus during viral entry [5], [6]. M2 protein is abundant on the surfaces of influenza A virus-infected cells but rare in mature virions [7], [8]. Ito *et al.* have demonstrated that the ectodomain of M2 protein (M2e), which contains 24 amino acids, is highly conserved among influenza A viruses [9]. Because of these properties, M2e has been considered as an attractive target for inducing cross-protection against different influenza A viruses subtypes [3]. It has been shown that the Abs specific to M2e could restrict influenza virus replication and reduce plaque size *in vitro* [8]. Passive immunization with these Abs reduced viral replication in the lungs of mice infected with influenza A virus [10]. Abs specific for M2e were rarely induced in human during natural influenza virus infection [11], [12] and in mice after experimental infection [13]. To overcome the low immunogenicity of M2 [14], [15], [16], a number of approaches have been attempted including fusion of M2 protein with carrier molecules like gluthation S-transferase,hepatitis B virus core (HBc), keyhole limpet hemocyanin (KLH), or *Neisseria meningitides* outer membrane protein complex (OMPC), or by co-administration with adjuvants such as flagellin and cholera toxin (CT) [17], [18], [19], [20], [21], [22], [23], [24], [25], [26].

The route of vaccine administration is critical for successful immunization [27]. Mucosal immune responses are important for the first line of defense because most microbial pathogens invade via mucosal surfaces [28]. It has been demonstrated that intranasal (i.n.) administration of M2 vaccines could induce better protection against influenza virus than parenteral immunization [18], [29]. However, i.n. administration of vaccines and certain adjuvants has met with safety issues associated with retrograde transport of immunogens or adjuvants to the central nervous system [30], [31].

Recently we have demonstrated that sublingual (s.l.) mucosa is an efficient site for the induction of broad-spectrum of immune responses [32]. S.l. administration of live or inactivated influenza virus induced Ab and T cell responses in the local mucosa of the respiratory tract and in the systemic compartment and protection of mice from lethal infection [33]. Importantly, unlike the i.n. route, s.l. immunization does not redirect vaccines to the CNS [32], [33].

In this study we examined the suitability of the s.l. immunization with M2-based vaccine for induction of broad protection against infection with different influenza virus subtypes in comparison with i.n., intradermal (i.d.) and intramuscular (i.m.) immunizations.

## Results

### Expression of soluble recombinant M2 proteins

**Figure 1:**
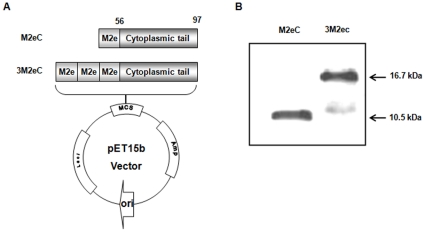
Construction of plasmids and purification of M2 proteins. (A) The synthetic M2eC or 3M2eC genes without hydrophobic region (amino acids 26–55) from PR8 virus were cloned into pET15b vector (B). The recombinant proteins expressed in *E. coli* were purified by His-tag affinity chromatography and detected by Western blot using M2e-specific monoclonal Ab, 14C2.

Since the deletion of amino acids 26–55 of M2 protein from A/Aichi/2/68 (H3N2) can improve solubility of the protein expressed from *E. coli* [17], gene without residues 26–55 of M2 protein from A/PR/8 (H1N1) was chemically synthesized and inserted into pET15b vector to express the target proteins as a fusion of his-tag at the N terminus (Fig. 1A). We prepared two constructs expressing one (M2eC) or three tandem copies (3M2eC) of M2e conjugated to C-terminus sequence of M2 protein. Each protein expressed from *E. coli* BL21 (DE3) strain was soluble and purified by His-Tag affinity chromatography. The endotoxin level of each protein was less than 5 EU/mg (data not shown). The purified proteins were confirmed by western blot using M2e-specific monoclonal Ab, 14C2 [8] (Fig. 1B). The result showed that M2eC and 3M2eC were 10.5 and 16.7 kDa, respectively.

### Immunogenicity of 3M2eC

**Figure 2:**
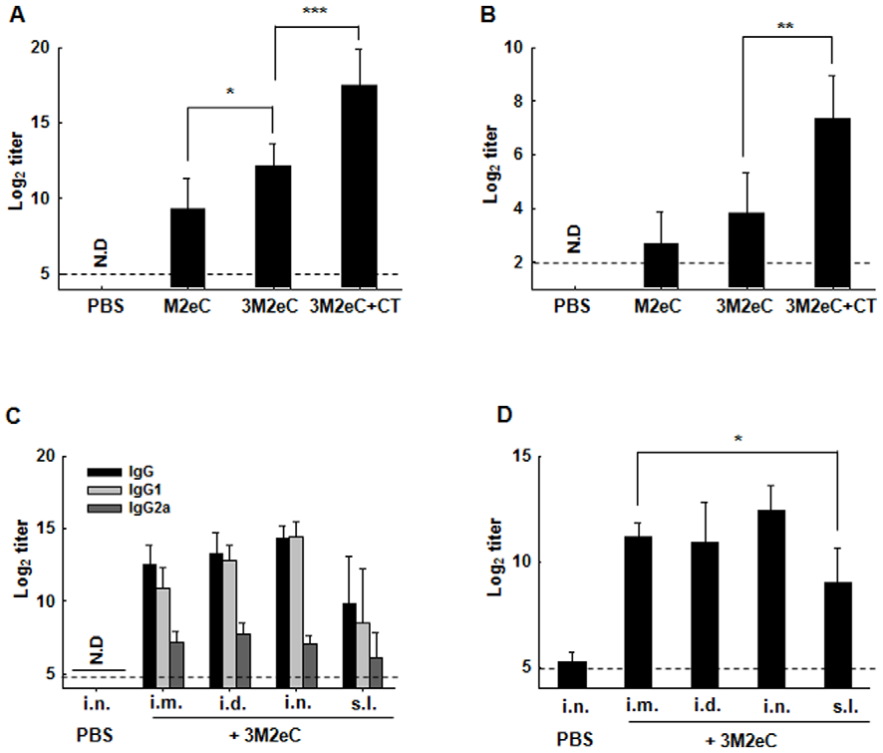
Immunogenicity of 3M2eC (A & B): BALB/c mice were immunized i.n. with 10 ug of M2eC, 3M2eC, or 3M2eC plus 2 ug of CT on day 0 and 14. Mice received PBS serve as control group. Sera and saliva were collected on day 14 after last immunization. Levels of M2e-specific IgG in sera (A) and IgA in saliva (B) were determined by ELISA. Ab levels induced by different immunization methods (C & D): BALB/c mice were administered on day 0 and 14 with 10 ug of 3M2eC protein plus 2 ug of CT for i.n. and s.l. immunizations or plus alum i.d. or i.m. immunizations. Sera were collected on day 14 after the last immunization. Ab and analyzed for M2eC-specific IgG subclasses by ELISA using 3M2eC protein (C) and M2e-specific IgG Ab by ELISA using M2e-expressing Hela cells (D). N.D., not detected. The dashed line shows the limit of detection. The results are expressed as the means+S.D. for the group (n = 5). The data are representative of three independent experiments. Significant differences were expressed as *, *P*<0.05, **, *P*<0.01, ***, *P*<0.005, respectively.

We first tested the immunogenicity of 3M2eC formulated with and without CT adjuvant when given intranasally. The immunogenicity of 3M2eC was compared to that of the single M2e protein. Each group of BALB/c mice was immunized i.n. twice with M2eC, 3M2eC alone, or 3M2eC mixed with CT (3M2eC/CT). As shown in Fig. 2A, mice immunized with 3M2eC in combination with CT showed significantly higher IgG titer than that seen in animals immunized with 3M2eC only. Mice immunized with M2eC developed significantly lower serum IgG response when compared to that induced in other two groups. Similarly, significant IgA titers were detected in saliva of mice immunized with CT adjuvanted 3M2eC as compared to that induced in saliva of other two groups (Fig. 2B). 3M2eC was more immunogenic than the M2eC construct and in consequence 3M2eC protein construct was used as a vaccine candidate for further experiments in this study.

### S.l. immunization with 3M2eC induced systemic immune responses

It has been shown that i.n. administration of M2-based vaccine is more effective than systemic routes for protection against influenza virus infection [26], [34]. However, i.n. immunization remains a safety concern because of potential retrograde transport of vaccine components to the CNS [30], [31]. In the earlier studies we demonstrated that s.l. administration with ovalbumin or inactivated virus induced cellular and humoral immunity comparable to i.n. immunization without redirection of antigens to the CNS [32], [33]. We further examined s.l. immunization with 3M2eC for induction of systemic immune responses in comparison with immunizations via i.n. and systemic routes (i.m. and i.d.). As shown in Fig. 2C, s.l. immunization with 3M2eC induced substantial M2e-specific serum IgG Ab response, although the level of Abs is lower than that induced by i.m., i.d., or i.n. immunization. Of note, i.n. immunization induced high level of specific IgG in plasma comparable to that induced by systemic immunizations. In addition, immunization with 3M2eC induced predominantly IgG1 as compared to IgG2a subclass (Fig. 2C).

To determine M2e-specific Ab response, full length tetrameric M2-expressing Hela cells [13] were used and Abs recognizing M2e on the surface of the cells were measured by ELISA. As shown in Fig. 2D, all the immunized mice exhibited high levels of M2e-specific Ab response. The level of M2e-specific Abs determined by ELISA with coated M2e was consistent with that determined by ELISA using 3M2eC protein.

### Protection against challenge with virus containing homologous M2e sequence

**Figure 3:**
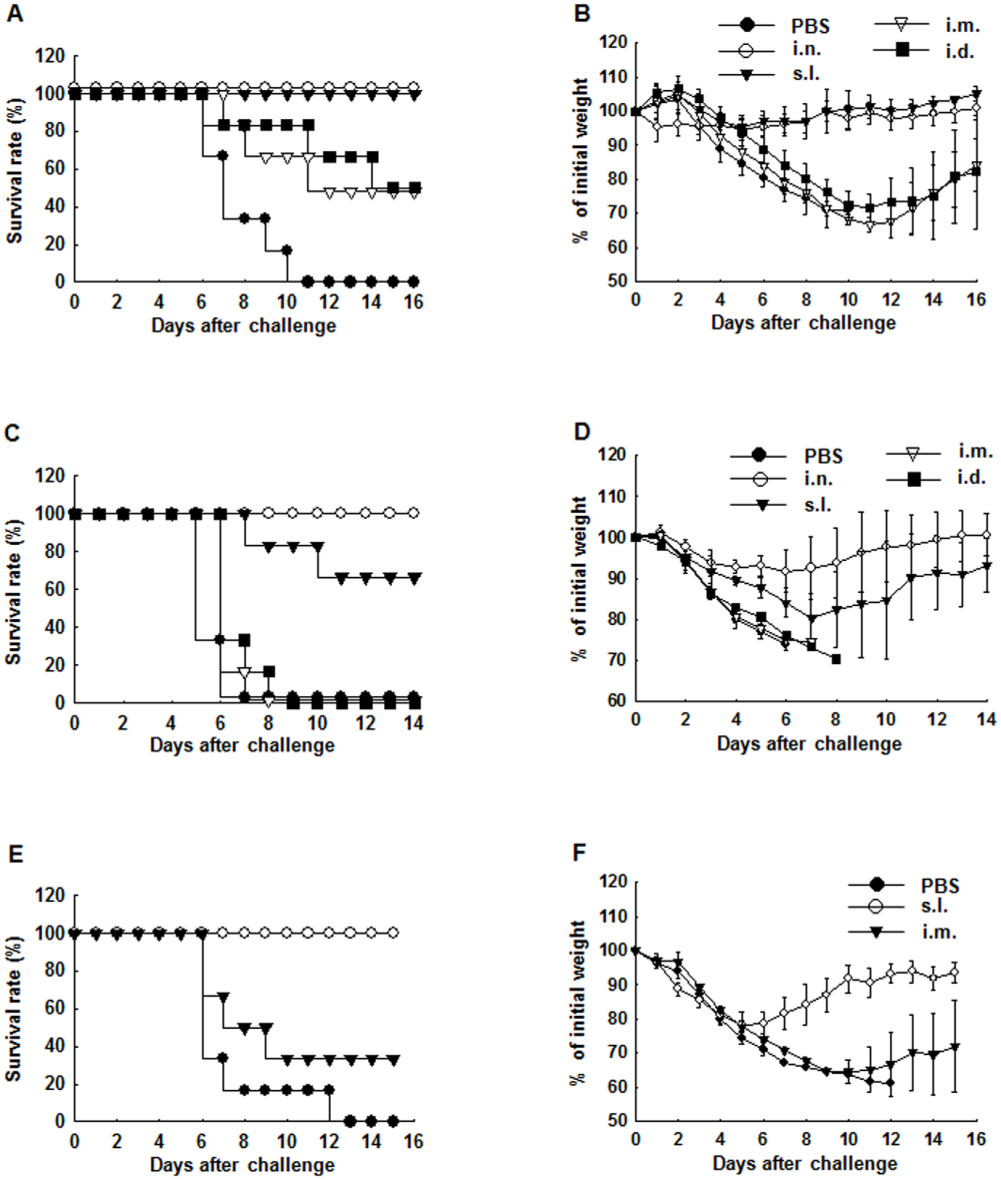
Cross-protection against infections with different influenza virus subtypes. Six-week-old female BALB/c mice (n = 6) were immunized twice with 10 ug of 3M2eC protein plus 2 ug of CT at 2 week intervals via i.n. or s.l., or with 10 ug of 3M2eC protein plus alum by i.d. or i.m.. They were challenged i.n. with 10 LD_50_ of mouse adapted PR8 strain (H1N1) at 3 weeks (A and B), A/Aquatic Bird/Korea/W81/05 virus (H5N2) at 3 weeks (C and D) or A/Philippine/2/82 (H3N2) virus at 5 weeks (E and F) after the last immunization. Survival rate and the body weight loss were monitored daily after the challenge. The results are expressed as the means+S.D. for the group.

We next evaluated the protective efficacy of the 3M2eC vaccine candidate against infection with influenza virus containing the same M2e sequence upon different immunization routes. BALB/c mice were immunized with 3M2eC twice at 2 week interval. Three weeks after the last immunization the mice were challenged i.n. with 10 LD_50_ of mouse-adapted A/PR/8 virus. As shown in Fig. 3A, only 50% of mice immunized via i.m. or i.d. route survived the challenge with lethal dose of PR8 virus challenge, while 100% of mice immunized sublingually or intranasally survived the challenge (Fig. 3A). As expected, none of the mice in the control unimmunized group survived the lethal infection. Significant body weight loss was observed in the groups of mice vaccinated via systemic routes (losing 33% and 28% of the initial body weight in groups of mice immunized via the i.m. and i.d. routes, respectively), while s.l. immunized mice lost less than 10% of initial body weight after the challenge (Fig. 3B). This result showed that s.l. administration with two doses of the 3M2eC protein provided complete protection against lethal influenza virus infection.

### Protection against challenge with virus containing heterologous M2e sequence

Although it is known that M2e is highly conserved among influenza A viruses [9], [35], we compared 10,551 M2e sequences of influenza A virus strains available from the U.S. National Center for Biotechnology Information (NCBI) with that of PR8 virus to examine homology of M2e sequence among the influenza A viruses (Table 1). We found that the most variable viruses contain 6 mismatched amino acids within 23 amino acids of M2e. To evaluate the efficacy of the 3M2eC vaccine candidate in induction of cross-protection, we selected a mouse adapted highly pathogenic A/Aquatic Bird/Korea/W81/05 (H5N2) virus that contains 6 mismatched amino acids against the M2e sequence of the PR8 virus (Table 2) [36] for challenge. While the systemic immunization routes failed to protect the mice against the lethal infection with H5N2 virus, the i.n. and s.l. immunization groups conferred 100% and 67% protection, respectively (Fig. 3C). Morbidity was minimally reduced in the mice immunized via i.n. route, while s.l. immunization group lost 20% of initial body weight and recovered on day 7 after challenge. In contrast, all mice in systemic immunization (i.m. and i.d.) groups lost more than 20% of initial weight and failed to recover. They all died within 8 days of the infection (Fig. 3D). We further examined cross-protective immunity against H3N2 virus, one of the seasonal strains that contain only 1 amino acid mismatched with M2e sequence of PR8 virus. As shown in Fig. 3E, i.m. immunization route induced partial cross-protection, whereas s.l. administration provided complete protection against challenge with lethal dose of mouse-adapted H3N2 virus. In addition, the s.l. immunization group showed rapid recovery from the weight loss compared to that seen in i.m. immunization group (Fig. 3F). Taken together, the results showed that although s.l. immunization with 3M2eC vaccine candidate induced lower level of Abs in plasma (Fig. 2C and D), it is superior to systemic immunizations in induction of protection against infections with virus containing identical or mismatched M2e sequence.

**Table 1:** Variations of M2e sequences among influenza A viruses.

No. of different amino acids	No. of influenza A virus strains
**0**	**968**
**1**	**1663**
**2**	**1955**
**3**	**4796**
**4**	**998**
**5**	**166**
**6**	**5**

Conserved amino acid																						
*	*	*	*	*	*	*	*	-	-	-	-	-	*	-	-	-	-	-	-	*	-	*
S	L	L	T	E	V	E	T	P	I	R	N	E	W	G	C	R	C	N	G	S	S	D

10,551 M2e sequences of influenza A virus strains were obtained from NCBI. These sequences were aligned with PR8-M2e sequence as a reference.

*denoted conserved sequence among the M2e sequences.

**Table 2:** Comparison of M2e sequences used in the present study.

Virus strain	Subtype	Amino acid sequence
A/PR/8	(H1N1)	SLLTEVETPIRNEWGCRCNGSSD
A/Aquatic bird/Korea	(H5N2)	SLLTEVETP**T**RN**G**W**E**C**K**C**SD**SSD
A/Philippine/2/82	(H3N2)	SLLTEVETPIRNEWGCRCN**D**SSD
A/CA/04/09	(new H1N1)	SLLTEVETP**T**R**S**EW**E**CRC**SD**SSD

M2e sequences were aligned with PR8-M2e sequence as a reference. The different amino acids from M2e sequence of PR8 virus are in bold.

### Protection against the 2009 pandemic influenza A virus (H1N1)

**Figure 4:**
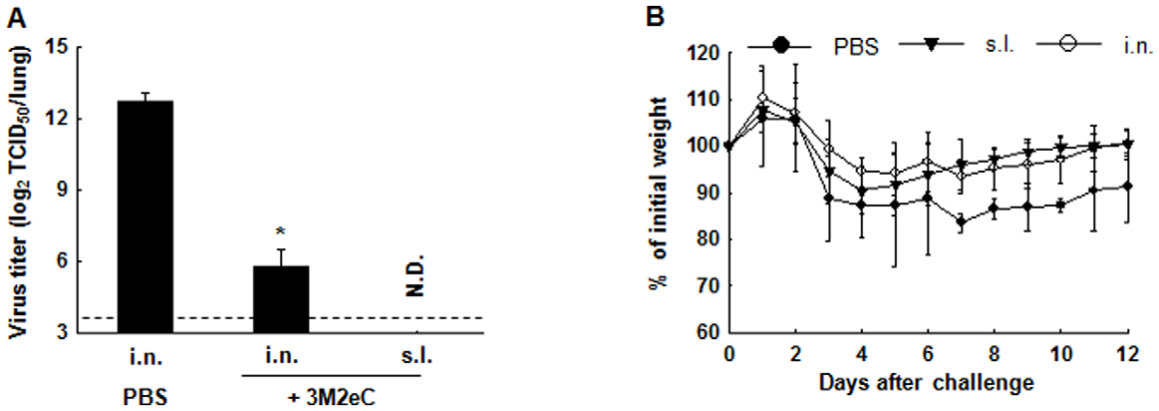
Protection against the 2009 pandemic influenza A virus (H1N1). Mice were immunized i.n. or s.l. with 3M2eC (10 ug) plus CT (2 ug) on days 0 and 14 and challenged by i.n. administration of A/CA/04/09 (H1N1) 5 weeks after the last immunization. (A) Virus titers in the lung tissue at day 5 after challenge were determined in embryonated chicken eggs. (B) Body weight was monitored daily after the viral challenge. The results are expressed as the means+S.D. for the group. Significant differences were expressed as *, *P*<0.05.

Because of the emergence of 2009 pandemic influenza virus and its establishment in human populations as a seasonal flu strain, we tested whether s.l. immunization with 3M2eC protein induces protection against challenge with 2009 pandemic H1N1 strain, which has 5 mismatched amino acids in the M2e sequence of PR8 virus. Mice were immunized sublingually with 3M2eC and challenged with A/CA/04/09 (H1N1) 5 weeks after the last immunization. Five days after the challenge, viral titers in the lungs were determined by EID_50_ assay. Significantly reduced viral titers were observed in groups of mice immunized via i.n. or s.l. route as compared to that in control group (Fig. 4A). Moreover, morbidity as assessed by loss of body weight was less in the immunized mice compared to the unimmunized control group (Fig. 4B). This result demonstrates that s.l. immunization with 3M2eC protein induced protection against infection with 2009 pandemic influenza virus in mice.

### S.l. or i.n. immunization with 3M2eC induced specific Ab responses in respiratory tract

**Figure 5:**
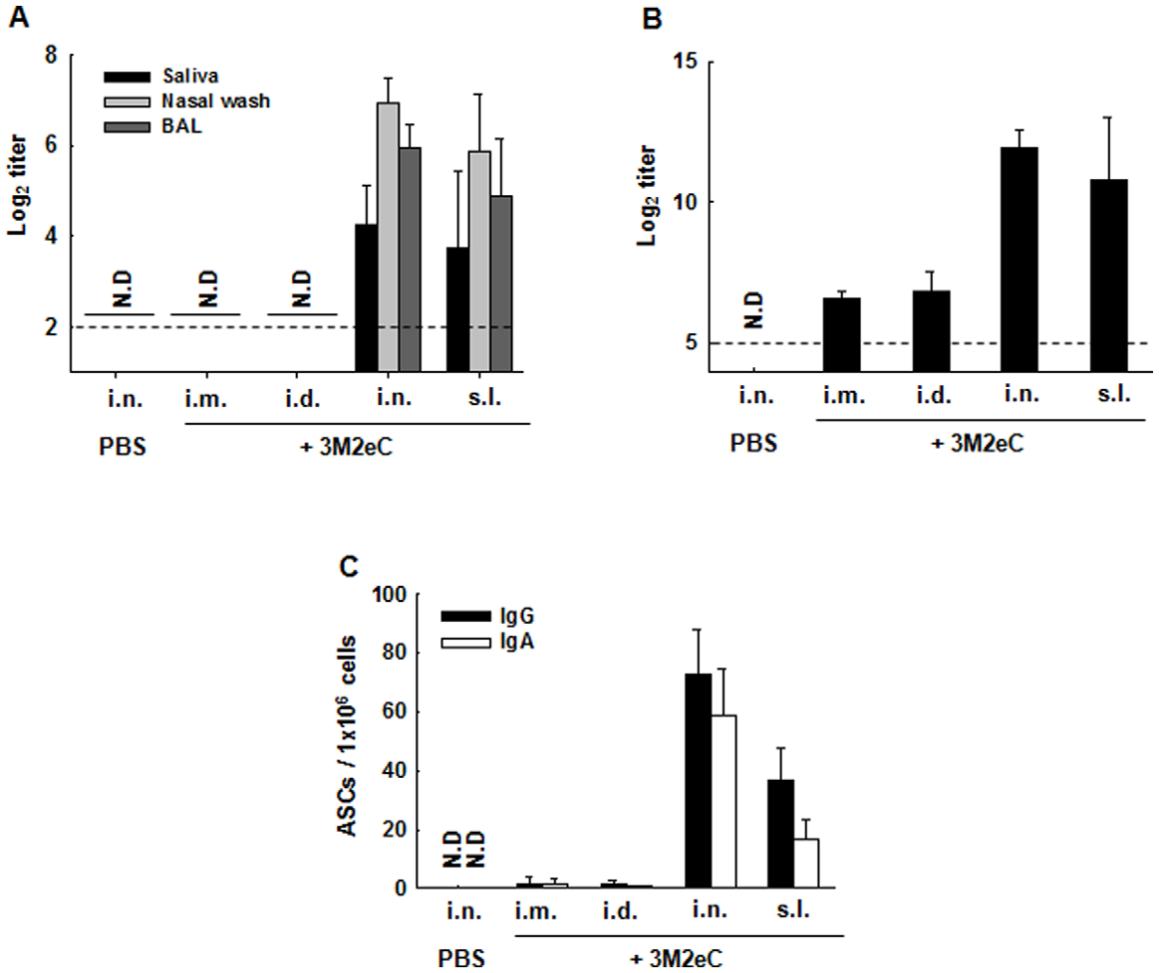
3M2eC-specific Ab levels in secretions and lung tissues. Mice were immunized with 10 ug of 3M2eC protein plus 2 ug of CT via i.n. or s.l., or with 10 ug of 3M2eC protein plus alum via i.d. or i.m. on day 0, 14, and 28. Saliva, nasal wash and BAL were collected two weeks after last immunization. M2e-specific IgA in the secretions (A) and M2e-specific IgG in BAL (B) were determined by ELISA using 3M2eC protein. (C) Number of M2e-specific IgG or IgA Ab secreting cells in the lung tissue at day 7 after last immunization was determined by ELISPOT using 3M2eC protein. N.D., not detected. The dashed line shows the limit of detection. The results are expressed as the means+S.D. for the group (n = 5). The data are representative of three independent experiments.

Since s.l. immunization with 3M2eC vaccine candidate induced better protection even with lower level of specific Abs induced in plasma as compared to systemic immunizations, we reasoned that s.l. immunization induced mucosal immune responses that are associated with protection. We determined levels of specific Abs in saliva, nasal wash and bronchoalveolar lavage (BAL) two weeks after the last immunization. We found that i.n. or s.l. immunization with 3M2eC induced significantly higher level of 3M2eC-specific IgA in saliva, nasal wash and BAL (*P*<0.05). In contrast, systemic immunization routes failed to elicit specific IgA (Fig. 5A). It has been recently shown that protection elicited by M2 vaccine is mediated by IgG-dependent alveolar macrophages in BAL [37]. We further examined 3M2eC-specific IgG level in BAL. Indeed, the levels of 3M2eC-specific IgG induced in mice immunized via s.l. or i.n. were significantly higher than those induced in the mice immunized via systemic routes (Fig. 5B). In addition, we enumerated the M2eC-specific Ab secreting cells (ASCs) in the lung tissues, the site of infection. We found significantly (*P*<0.05) high numbers of 3M2eC-specific IgG and IgA ASCs in the lung tissue of the mice immunized via i.n. or s.l. route (Fig. 5C), while only a few ASCs were observed in the lung tissues of i.m. or i.d. immunized mice. The results suggest that Ab responses induced in the lungs upon mucosal immunization with M2-based vaccine are important for protection against influenza virus infection.

## Discussion

In this study, we demonstrate that s.l. administration of a recombinant trimeric M2e protein construct adjuvanted with CT induces protection against a lethal challenge with wild-type influenza virus. Protection conferred by s.l. M2e vaccine construct was superior to that induced by systemic immunizations. Although the level of M2e-specific serum IgG Ab after s.l. immunization was lower than those seen after systemic immunizations, s.l. immunization induced higher M2-specific Ab titers in saliva, nasal wash and BAL, as well as M2-specific ASCs in lung tissues. Our results clearly suggest that specific Abs induced in mucosa-associated tissues after s.l. immunization are important for protection in M2-based vaccine against infection with influenza A viruses.

M2e, being relatively highly conserved among the influenza A viruses, has been considered a most promising influenza vaccine antigen [9]. A number of strategies have been developed to induce cross-protection using M2e-based vaccines [18],[24],[25]. The most common of these strategies involves systemic such as s.c. or i.m. administration of the antigen but protection has often been rather limited [14], [15]. In keeping with these observations, our study indicates that systemic administration with two doses of 3M2eC conferred partial and in some cases no protection against challenge with different virus subtypes. In most studies, systemic immunization with at least three doses of M2-based vaccine was required to achieve full protection [19], [21], [38], [39]. Other studies have shown that systemic administration of two doses of M2-based vaccine induced protection against challenge with relatively low (1 LD_90_ or 4 LD_50_) doses of wild type influenza virus [24], [40].

In our study, two doses of 3M2eC via systemic routes conferred partial or no protection against the challenge with 10 LD_50_ of influenza A viruses. However, i.n. immunization with two doses of 3M2eC conferred full protection against challenge with different influenza A virus subtypes. These findings support the observations that the i.n. route, a mucosal route, is superior to systemic administration routes for promoting cross-protective immunity in mice [18], [26], [34]. Importantly, our study demonstrates for the first time that s.l. immunization with just 2 doses of M2-based vaccine candidate induced broad protection against challenge with relatively high dose of lethal influenza A virus.

The mechanisms by which Abs against M2e mediate cross-protection are not fully understood. In this study, we show that M2e-specific Ab responses are induced in the lungs after mucosal (s.l. or i.n.) rather than systemic (i.m. or i.d.) administration, and these responses are associated with protection against influenza virus infection. These results further support recent findings that anti-M2e IgG Abs are involved in protection through interaction with Fc receptors expressed on alveolar macrophages [37]. Indeed, we found significant levels of anti-M2e IgG induced in BAL and ASCs in the lungs after s.l. or i.n. but not systemic immunization.

Several studies have reported that Abs to N-terminus of M2e inhibit replication of influenza A virus [11] and that adoptive transfer of monoclonal Abs to an epitope located between position 1 and 10 on the N-terminus sequence of M2e protect against influenza A virus challenge [41], [42]. In our study, protection against highly heterologous influenza viruses upon s.l. immunization with M2-based vaccine is probably due to induction of Abs to N-terminus (position 1–10) of M2e sequence that is identical to the sequences of the viruses used for challenge in our study.

We showed that the i.n. route conferred complete protection against homologous and heterologous challenges, suggesting that i.n. administration is potent to induce protection against multiple subtypes of influenza A virus. However, it has remained a safety issue for human use due to accumulation of antigens to CNS [30], [31]. In contrast with i.n., the s.l. route is considered to be safe, since redirection of an antigen to the CNS does not occur [32], [33]. Our previous study has demonstrated that s.l. immunization with ovalbumin induced significant mucosal and systemic immune responses, as well as cytotoxic T cell response in lung tissue [32]. In addition, s.l. administration with inactivated influenza virus provided protection against influenza virus challenge without redirecting the immunogen to the CNS [33]. However, it is not yet clear how s.l. immunization could induce Ag-specific Ab responses in the lung mucosa.

Recently, there has been some concern regarding the possible emergence of a new influenza pandemic by reassortment between animal and human viruses [43]. In fact, new pandemic H1N1 virus occurred worldwide in 2009 resulting in significant morbidity and mortality [44]. After the first human infection in Hongkong [45], highly pathogenic avian influenza virus H5N1 (HPAIV) caused number of human infections with a death rate of more than 50% and remains a global threat [46]. For pandemic influenza preparedness development of universal influenza vaccines against various subtypes is urgent. Our vaccination strategy of combination of s.l. immunization, a safe mucosal route, with M2-based vaccine provided broad protection against different influenza. Our vaccination strategy offers a new tool for control of influenza outbreaks including future pandemics. Of note, the protection against the wide range of the viruses containing mismatched amino acids ranging from 0 to 6 out of 23 amino acids of M2e from PR8 strain was observed.

During a pandemic, the availability and rapid mobilization of medical care personal is critical for effective mass vaccination [47]. Since s.l. mass immunization could be implemented without requiring trained healthcare personnel, this approach may be deployed under complex emergency situations such as during the early stage of an epidemic outbreak.

In conclusion, our results demonstrate that s.l. immunization with an M2e-based vaccine formulation is efficacious against experimental infection in mice. These findings may offer an approach to control epidemic and pandemic influenza infections.

## Materials and Methods

### Construction of plasmids expressing M2eC or 3M2eC protein

A gene (Fig. 1A) encoding three tandem copies of M2e conjugated to C-terminus sequence of M2 protein without residues 26–55 from influenza A/Puerto Rico/8/34 (H1N1) virus was chemically synthesized by Bio S&T Inc. (Canada). The gene has an Nde I site between 2^nd^ and 3^rd^ M2e region. For the plasmid expressing 3M2eC, the gene was digested with Xho I and BamH I and inserted into the bacterial expression vector pET15b (Novagen, Madison, WI) to express as a fusion of his-tag at the N terminus, resulting in the plasmid pET15b-3M2eC. For the construct expressing M2eC protein, which has one M2e domain, the synthesized gene was digested with Nde I and BamH I, and then inserted into pET15b vector.

### Expression and purification of M2eC or 3M2eC proteins

*E. coli* BL21 (DE3) strains (Novagen) transformed with these plasmids were grown overnight at 37°C in Luria-Bertani (LB) medium supplemented with 100 ug/ml of ampicillin. The overnight culture was transferred into fresh LB medium and cultured at 37°C while shaking at 180 rpm until OD_600_ of 0.6∼0.8. Each protein expression was induced by adding IPTG (isopropyl β-D thiogalactoside) to a final concentration of 0.5 M for 4 hrs and the cells were harvested by centrifugation at 6,000 rpm for 10 min. The cell pellets were suspended in binding buffer (20 mM Tris, 0.5 M NaCl, pH 7.9) and disrupted by sonication on ice. Then, the soluble and insoluble fractions were separated by centrifugation for 40 min at 20,000 rpm. The soluble fractions were applied to a Talon metal affinity column (Clontech, Palo Alto, CA). The columns were washed with binding buffer containing 20 mM imidazole, and then the proteins were eluted by an elution buffer (300 mM imidazole, 20 mM Tris, 0.5 M NaCl, pH 7.4), followed by desalting with PD3 column (Amersham, IL, USA). The purified proteins were treated with 1% Triton X-114 to remove endotoxin and incubated with rocking for 30 min at 4°C, followed by incubation in a 37°C water bath for 20 min. The phases containing endotoxin were separated by centrifugation at 13,000 rpm for 5 min. This cycle was repeated five times. Each protein was incubated with SM-2 beads (Bio-Rad, Hercules, CA) for 2 hrs at 4°C to remove residual Triton X-114 and filtered through spin-X column (Costar, Lowell, MA). The endotoxin level of each protein was measured by the limulus amebocyte lysate (LAL) assay kit according to the instructions (Lonza, Switzerland). Endotoxin levels of the proteins were less than 5 EU/mg. The purified proteins were electrophoresed on 15% SDS-PAGE and the protein bands were visualized by staining with Coomassie Brilliant Blue. The protein concentration was determined by Bradford protein assay kit (Bio-Rad). The purified proteins were stored at −80°C.

### Western blot

The purified proteins were separated on 15% SDS-PAGE and the gel was transferred onto a nitrocellulose membrane (Schleicher & Schuell, Germany) by using a semi-dry transblot apparatus (Bio-Rad). The membrane was blocked with Tris-buffered saline (TBS) containing 5% skim milk for 30 min at room temperature and incubated with M2e specific Ab (14C2) [8] at 1∶1,000 dilution in TBST (TBS and 0.05% Tween 20) containing 5% skim milk for 1 hr at room temperature. After washing with TBST, the membrane was probed with Goat-anti mouse IgG conjugated with horseradish peroxidase (Santa Cruz biotechnology, Delaware, CA) at 1∶3,000 dilution in TBST containing 5% skim milk for 1 hr at room temperature and detected with an ECL kit (Amersham).

### Mice and immunization

Specific pathogen free, female BALB/c mice aged 6 weeks were purchased from Orient Bio Inc. (Korea). All mice were maintained under specific pathogen-free conditions and all studies were approved by Institutional Animal Care and Use Committees (IACUC) at Yonsei University (2010-00-32619), Chungbuk University (BLS2011-0003) and International Vaccine Institute (2010-017). In order to compare immune response between 3M2eC and M2eC proteins, five mice per group were anesthetized with ketamine and immunized i.n. with 20 ul containing 10 ug of M2eC, 3M2eC alone, or with 2 ug of CT (LIST BIOLOGICAL LABS INC. Campbell, CA) on day 0 and 14. To compare protective immunity depending on the route, mice were immunized with 10 ug of 3M2eC protein plus 2 ug of CT by i.n. or s.l., or with 10 ug of 3M2eC plus alum by i.d. or i.m. on day 0 and 14. For i.n. immunization, total 20 ul of prepared vaccines were administered into each nostril of the anesthetized mice. For s.l. immunization, the anesthetized mice were immunized with 15 ul of vaccines underneath the tongue using a pipette. Following s.l. immunization, mice were maintained with heads placed in ante flexion for 30 min. For i.d. immunization, the anesthetized mice's chests were shaved. The needle was inserted into the skin nearly parallel to the plane of the skin and 100 ul of vaccines were administered per mouse. For i.m. immunization, 100 ul of vaccines were injected into the thigh muscles of mice.

### Sample collection

Sera and mucosal samples were collected on days 13 or 14 after the last immunization. Blood was collected from the retro-orbital plexus, incubated at room temperature for 30 min and the sera were obtained from the blood by centrifugation for 10 min at 13,000 rpm. Saliva samples were obtained after inducing salivary gland secretion by i.p. injection of pilocarpine (100 ug per animal) (Sigma, St. Louis, MO). For BAL samples, the mice were dissected to expose the trachea and then IV catheter (BD Biosciences, San Jose, CA) was inserted into a small nick of the trachea. BAL samples were collected by repeated flushing and aspiring with 500 ul of PBS into the lungs. Nasal washes were collected by flushing with 50 ul of PBS two times through the nasal cavity. The samples were stored at −80°C until used.

### ELISA

Ab titers were measured by enzyme-linked immunosorbent assay (ELISA) using serum or mucosal samples from each mouse (n = 6). The 96-well ELISA plates (Nunc, Roskilde, Denmark) were pre-coated with 100 ul of 3M2eC protein (2 ug/ml) in 50 mM Sodium bicarbonate buffer (pH 9.6) overnight at 4°C. After blocking with PBS containing 5% skim milk for 1 hr at room temperature, 100 ul of serial 2- or 3-fold diluted samples in blocking buffer were added to each well and incubated for 1 hr at 37°C, followed by addition of 1∶3,000 diluted horseradish peroxidase-conjugated goat anti-mouse IgG, IgG1, IgG2a, or IgA (Santa Cruz biotechnology). After incubation for 1 hr at room temperature, 100 ul of peroxidase substrate tetramethylbenzidine (TMB) (Millipore, Bedford, MA) was added to each well. The reaction was stopped by adding 0.5 N HCl. The absorbance at wavelength 450 nm was recorded by an ELISA reader (Molecular Devices, Sunnyvale, CA). The endpoint titer was determined by O.D. cut-off values of 0.2.

### M2 expressing Hela cell-based ELISA

M2 expressing Hela cells in RPMI1640 containing doxycycline (0.5 ug/ml) were dispensed at 1×10^5^ cells per well into 96-well plates. Next day, the plates were fixed by addition of 80 ul of 80% acetone, followed by washing with PBS for three times. Serum IgG titers to M2 expressing Hela cells (M2e) were determined by ELISA as above.

### ELISPOT assay

Mice (n = 5) were immunized on day 0, 14, and 28 as described above. On day 7 after the last immunization, the mice were anesthetized and the lungs were removed into RPMI medium. To obtain the lung cell suspension, the tissues were minced with scissors and incubated for 1 hr at 37°C in RPMI medium containing 0.5 mg/ml collagenase D (Roche Applied Science, Indianapolis, IN) and 100 µg/ml of DNase (Sigma-Aldrich). Following incubation, the cell suspensions were obtained by passing gently through a 100 um Falcon cell strainer (BD Labware). The number of M2-specific ASCs in the lung cell suspensions was calculated by using ELISPOT assay. 96-well nitrocellulose microplates (Millipore) were coated with 3M2eC (10 ug/ml) in PBS overnight at 4°C. Next day, the plates were blocked with RPMI-1640 (Cambrex, Walkersville, MD) containing 10% FBS (complete medium) for 30 min at 37°C in 5% CO_2_ incubator. The lung cell suspensions were transferred to ELISPOT plates at 2-fold dilutions, followed by addition of HRP conjugated goat anti-mouse IgG or IgA (1∶500) (Southern Biotechnology Associates, Birmingham, AL). After incubation for 4 hrs in a 37°C, 5% CO2 incubator, the plates were extensively washed with PBS, PBS-T, water, sequentially. Spots were developed by addition of AEC-H_2_O_2_ chromogenic substrate (Sigma-Aldrich) and counted by using an ELISPOT reader (Molecular devices)

### Virus challenge

For the homologous challenge, the immunized mice were anesthetized and then challenged i.n. with 10 LD_50_ (40 TCID_50_) of mouse adapted influenza A/Puerto Rico/8/34 (H1N1) virus three weeks after the final vaccination. For the heterologous challenge, the immunized mice were infected with 10 LD_50_ (89 TCID50) of mouse adapted A/Aquatic Bird/Korea/W81/05 (H5N2) virus three weeks after the final vaccination or with 10 LD_50_ (320 PFU) of mouse adapted A/Philippine/2/82 (H3N2) virus five weeks after the last vaccination. The mice were monitored daily for weight loss and survival rate following the viral challenge. Survival rate was determined by death or a cut-off of 25% in body weight loss at which point animals were euthanized.

### Virus Titers in lung tissues

Mice (n = 5) were immunized i.n. with 20 ul containing 10 ug of M2eC, 3M2eC alone, or with 2 ug of CT as adjuvant, administered 2 weeks apart. Five weeks after last vaccination, mice were i.n. challenged with 30 ul (3×10^3^ TCID_50_) of the A/CA/04/09 (H1N1) virus. Mice were continuously observed for 12 days post infection (dpi). Lung tissue samples of mice were collected at 5 dpi and homogenized in PBS containing antibiotics. Tissue homogenates were centrifuged at 12,000× g and supernatants were transferred to new tubes. All samples were immediately serially diluted 10-fold and then inoculated into 11-day-old embryonated chicken eggs for virus titration as computed by the Reed and Muench method with results expressed as (EID_50_/ml) [48].

### Statistical analysis

Statistical tests were performed by using Student's *t* test. A *P* value of less than 0.05 was considered significant.
